# Effect of a unilateral hind limb orthotic lift on upper body movement symmetry in the trotting horse

**DOI:** 10.1371/journal.pone.0199447

**Published:** 2018-06-21

**Authors:** Jodi Vertz, Diana Deblanc, Marie Rhodin, Thilo Pfau

**Affiliations:** 1 Healthy Hoofcare, Albuquerque, NM, United States of America; 2 University of Edinburgh, The Royal (Dick) School of Veterinary Studies, Edinburgh, United Kingdom; 3 Performance Equine Veterinary Services, Peralta, NM, United States of America; 4 Department of Anatomy, Physiology and Biochemistry, Swedish University of Agricultural Sciences, Uppsala, Sweden; 5 Department of Clinical Science and Services, The Royal Veterinary College, London, United Kingdom; University of Minnesota, UNITED STATES

## Abstract

In trotting horses, movement asymmetry is associated with ground reaction force asymmetry. In humans, limb length differences influence contralateral force production. Here we investigate whether horses, in immediate reaction to limb length changes, show movement asymmetry adaptations consistent with reported force differences. Aim of this study was to quantify pelvic and compensatory head and withers movement asymmetry as a function of limb length changes after application of orthotic lifts. In this experimental study movement asymmetry of eleven trotting horses was calculated from vertical displacement of poll, withers, sacrum and left and right tuber coxae with inertial sensors. Horses were assessed in-hand under 5 conditions (all with hind limb boots): without orthotic lifts, and with a 15mm or 30mm orthotic lift applied to the left hind or right hind. A linear mixed model investigated the influence of orthotic lift condition (P<0.05, pairwise posthoc Bonferroni correction). Pelvic movement asymmetry showed increased pelvic downward movement during stance of the shorter limb and increased pelvic upward movement during and after stance of the longer limb (P<0.001) with asymmetry changes of 3-7mm (4-10mm) for 15mm (30mm) lifts. Hip hike (tuber coxae movement asymmetry) was unaffected (P = 0.348). Head and withers movement asymmetry were affected less consistently (2 of 3 respectively 1 of 3 head or withers parameters). The small sample size of the study reduced generalizability, no direct force measurements were conducted and only immediate effects of orthotic lifts were assessed with no re-assessments days or weeks after. Conclusions about mechanical consequences (weight bearing, pushoff) are based on published movement-force associations. Pelvic movement asymmetry with an artificial change in limb length through application of an orthotic lift indicates increased weight support with the shorter limb and increased pushoff with the longer limb. This may be of relevance for the management of horses with different hoof shapes between contralateral limbs, for example some chronically lame horse.

## Introduction

Parameters describing asymmetry of upper body movement in trot–commonly referred to as head nod and hip hike–are often used for the purpose of detecting the presence and quantifying the severity of lameness in the horse [1,2], a clinical sign suggesting the necessity of further veterinary assessment for any indications of a painful or mechanically restricting defect being the cause of the gait abnormality. When a horse with hind limb lameness trots, the asymmetrical movement of the pelvis–which can be quantified by comparing the two minima (and/or maxima) in vertical position [3]–is associated with differences in ground reaction force production between the left and right hind limb stance phases [4]. Similar associations have been identified between asymmetrical head movement and differences in forelimb force production [5]. This ‘simple’ association between upper body movement asymmetry and asymmetrical force production provides a means of interpreting the kinematics of lameness in the framework of basic mechanics. For example, measuring a decrease in movement asymmetry after diagnostic analgesia [6,7] or a (temporary) increase in asymmetry after a flexion test [8,9] can be assumed to lead to more or less symmetrical force production between contralateral limbs. Hence these interventions are used by veterinarians to ascertain whether or not the clinical sign of movement asymmetry is likely linked to an underlying pathological condition.

In addition to shifting force to the contralateral, unaffected limb, hind limb lame horses also redistribute force from the hind limbs to the forelimbs and vice versa in forelimb lame horse [10,11]. Consequently, some clinically lame horses show concurrent head and pelvic movement asymmetry [12,13]. The ‘law of sides’ [13] relates a concurrent ipsilateral head and pelvic asymmetry to an increased likelihood of a primary hind limb lameness and a contralateral asymmetry to a primary forelimb lameness. Adding measurement of withers movement asymmetry into a gait assessment has shown good discriminative potential between induced forelimb lameness (head and withers movement asymmetry same-sided) and induced hind limb lameness (head and withers movement asymmetry of opposite direction) [14]. Synchronous measurement of head, withers and pelvic movement hence appears to be a workable compromise between in-field applicability (measurement with cameras or inertial measurement units rather than direct measurement of force with force platforms) and providing sufficiently accurate and precise measurements [15,16] allowing insights into the fundamental mechanics related to force asymmetry in lame horses. In addition to underlying pain, ‘mechanical defects’ [17] are referenced as causes for horses showing lameness. The notion of the lameness being a clinical sign [17] indicates the need for further veterinary investigations establishing whether further attention is needed. A previous study of 222 horses in training showed motion asymmetries in 73% of horses during straight line trot [18]. However, with technological advances in quantifying subtle gait asymmetries, the question arises whether all measurable movement asymmetries need further veterinary attention [19]. Whether inherent motor laterality or conformational asymmetries, in particular limb length differences, can result in motion asymmetries affecting vertical trunk displacement in horses has not been investigated.

In humans, limb length discrepancies lead to increased weight bearing with the shorter limb and increased pushing off (propulsion) with the longer limb [20,21]. In horses, manipulation of limb length is for example of interest for correcting force asymmetries in the presence of asymmetrical feet [22], which may be relevant for rehabilitation regimens in horses with differently shaped hooves. It may also be interesting to contrast the effects of manipulating limb length to the effect of toe or heel wedges in the clinical lameness examination [23].

Aim of this study is to quantify the immediate effects of artificial changes in distal limb conformation (a simple mechanical change), in particular hoof height discrepancies creating a limb length difference between contralateral hind limbs, on movement asymmetry. We hypothesize that, in the presence of a unilateral hind limb orthotic lift, i.e. an artificial lengthening of one limb, pelvic movement asymmetry will be consistent with signs of reduced weight bearing with the longer limb (the limb with the orthotic lift) and with signs of reduced pushoff with the shorter limb (the limb contralateral to the orthotic lift). It is difficult to hypothesize the direction of any compensatory head and withers movement asymmetries or the direction of tuber coxae movement differences due to the hypothesized mixed effect on pelvic movement asymmetry and as such the investigation of head and withers movement asymmetries has explorative character.

## Materials and methods

This study was approved by the Royal (Dick) School of Veterinary Studies Ethical Review Committee. Horse owners gave written informed consent for participation in the study.

### Animals

Eleven riding horses not known to be clinically hind limb lame at the time of data collection were included in the study (5 mares, 6 geldings, 10 to 25 years of age (average 16 years), 4 Paint horses, 2 Quarter horses, 2 Arabians, 1 Appaloosa, 1 Warmblood, 1 Thoroughbred. All horses were in good body condition score (BCS 3–5, [24]).

### Interventions

**Fig 1:**
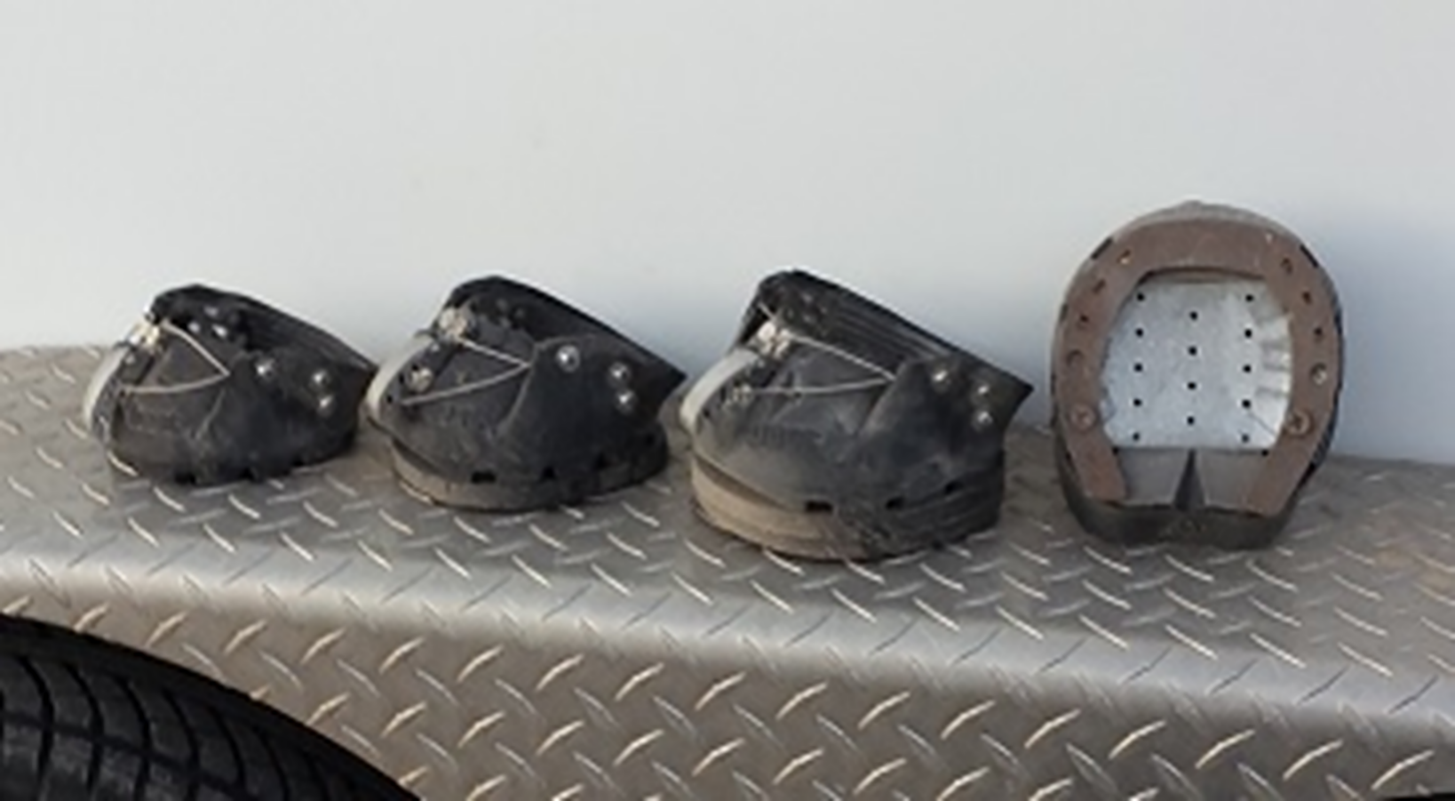
Photo of hind limb boots used in the study. From left to right: Easycare original easyboot without orthotic lift, boot with 15mm lift, boot with 30mm lift, boot with steel shoe used in contralateral hind limb when the 30mm lift was used.

Easycare original easyboot (EasyCare, Inc. Tucson, AZ, US) hoof boots were used to apply ‘orthotic lifts’ (i.e. an artificial lengthening of the limb by increasing hoof height) to one hind hoof at a time (Fig 1). The lifts were made from 15mm thick Castle (Castle Plastics, Leominster, MA, US) double nail pads. Lifts of 15mm height and 30mm height (two pads) were applied. A steel sliding plate horse shoe was added to the bottom of the non-lift boot during the 30mm variable trials in order to keep the weight difference between contralateral limbs the same for trials with 15mm and 30mm. This resulted in the effective height difference between the boot with the 30mm lift and the non-lifted boot to be reduced to 23mm.

Horses were assessed under five conditions:

- ‘no lift’: boots fitted to both hind limbs (without any additional pads)
- 15L: boots on both hind limbs. One 15mm pad added to the left hind (LH) boot
- 15R: boots on both hind limbs. One 15mm pad added to the right hind (RH) boot
- 30L: boots on both hind limbs. Two 15mm pads added to the LH boot, steel sliding plate fitted to RH boot (effective difference 23mm).
- 30R: boots on both hind limbs. Two 15mm pads added to the RH boot, steel sliding plate fitted to LH boot (effective difference 23mm).

### Gait analysis

Quantitative gait analysis was performed by the same licensed veterinarian (DD) with five inertial measurement units (IMUs, MTw 2^nd^ generation, Xsens, Enschede, The Netherlands) attached to poll, withers, sacrum (between tubera sacrale) and left and right tuber coxae. Horses were assessed in familiar surroundings and trotted in-hand (sand or dirt surface) subjectively aiming for collection of a minimum of 25 strides per condition.

Data processing followed published procedures (implemented in MATLAB, The Mathworks INC, Natick, MA, US) and involved high pass filtering, rotation into horse-gravity based reference frame and numerical double integration [15] followed by stride segmentation making use of pelvic rotation and vertical velocity [25]. Movement asymmetry measures were then calculated from minima and maxima identified in vertical displacement traces [3]. Here we calculate differences between minima (head: HDmin, withers: WDmin, mid pelvis: PDmin), differences between maxima (head: HDmax, withers: WDmax, mid pelvis: PDmax) and differences between upward amplitudes (head: HDup, withers: WDup, mid pelvis: PDup) as well as differences between upward movement amplitudes between left and right tuber coxae (HHD). Median asymmetry values over all strides collected for each condition were tabulated according to horse, orthotic lift condition (no lift, L15, R15, L30, R30) together with the number of strides and the average stride time (S1 Table for complete data set).

For illustrative purposes, signs of asymmetry parameters (HDmin, HDmax, HDup, PDmin, PDmax, PDup) are interpreted in comparison to displacement traces of horses with induced forelimb and hind limb lameness [1,3] and labelled as L for signs corresponding to traces observed in left lame and R for signs corresponding to traces observed in right lame horses. Withers movement was labelled with the same convention as head movement. HHD was labelled based on the observation of increased movement amplitude of the tuber coxae on the lame side [2]. This is simply a judgement of the direction of asymmetry allowing to use the terms left-sided and right-sided asymmetry with reference to published traces of horses with left-sided and right-sided (induced or natural) lameness [1–3].

### Statistical analysis

A mixed linear model was implemented (SPSS, IBM, Armonk, NY, US) for each movement asymmetry variable with horse as random factor, stride time as covariate and condition as fixed factor. Ten movement asymmetry variables (HDmin, HDmax, HDup, WDmin, WDmax, WDup, PDmin, PDmax, PDup and HHD) were investigated. Histograms of model residuals were inspected visually for normal distribution. Significance level was chosen at P<0.05 and when a significant overall effect was found, pairwise comparisons were conducted with Bonferroni post hoc corrections applied.

Box plots were created to illustrate movement asymmetry parameters for the different orthotic lift conditions (‘no lift’, 15L, 15R, 30L, 30R). Plots showing median values (circled dots), 25^th^ and 75^th^ percentile (interquartile range, IQR, thick lines), whiskers (thin lines) extending to median +/-1.5*IQR and extreme values outside the area covered by the whiskers. In order to study magnitude and direction of effects of asymmetry, estimated marginal means (and their confidence intervals) from the mixed model analysis were evaluated.

## Results

### Data collection summary

Data collection from the 11 horses resulted in median gait asymmetry values calculated across a total of 1365 strides (average 25.8, minimum 13, maximum 38). Data collection in ten horses included all five conditions. In the remaining horse, only the ‘no lift’, 30L and 30R conditions were successfully collected. Stride time on average across all 53 trials of 11 horses was 696.1ms (minimum 624ms, maximum 805ms). Absolute values of movement symmetry variables for head and pelvis displacement were larger than the previously described thresholds of >8mm for head movement and >4mm for pelvic movement (thresholds from [26] adapted to the inertial sensor system used here with equations in [27]) in 7 (out of 11) horses for HDmin, 5 for HDmax, 6 for PDmin and 7 for PDmax.

### Effect of unilateral orthotic hind limb lift

The results of the mixed linear models for each movement asymmetry parameter are summarized in Table 1. None of the models revealed a significant influence of stride time (all P> = 0.138). Without application of an orthotic lift (condition ‘no lift’), the estimated marginal means show on average across horses comparatively small amounts of movement asymmetry from 3mm left-sided asymmetry for WDup to 2mm right-sided asymmetry for PDmax and HHD (Table 2). The 95% confidence intervals typically span a range of 10mm to 16mm for pelvis and withers related movement asymmetry and up to 29mm for head movement asymmetry indicating a fair amount of variation between horses.

**Table 1:** Results of mixed model analysis of pelvic, head and withers movement asymmetry with horse as random factor, stride time as covariate and orthotic lift condition as fixed factor. Level of significance was set to P<0.05 and pairwise Bonferroni comparisons conducted for asymmetry parameters found to be significantly affected by orthotic lift condition.

asymmetry parameter	condition	stride time	pairwise comparison (Bonferroni corrected P-values)	
**PDmin**	<0.001	0.656	0.047	‘no lift’ <-> L15
			0.001	‘no lift’ <-> L30
			<0.001	‘no lift’ <-> R30
			0.001	L15 <-> R15
			<0.001	L15 <-> R30
			<0.001	R15 <->L30
			0.012	R15 <->R30
			<0.001	L30 <-> R30
**PDmax**	0<0.001	0.138	0.029	‘no lift’ <-> R30
			0.010	L15 <->R15
			0.001	L15 <-> R30
			0.007	L30 <-> R30
**PDup**	0.020	0.293	0.012	L30 <-> R30
**HHD**	0.348	0.335	not applicable	not applicable
**HDmin**	0.096	0.152	not applicable	not applicable
**HDmax**	<0.001	0.677	0.001	‘no lift’ <-> R30
			0.010	L15 <-> R30
			0.039	R15 <-> R30
			<0.001	L30 <-> R30
**HDup**	<0.001	0.235	0.003	‘no lift’ <-> R30
			0.004	L15 <-> R30
			<0.001	L30 <-> R30
**WDmin**	0.003	0.278	0.011	L15 <-> R30
			0.011	L30 <-> R30
**WDmax**	0.102	0.741	not applicable	not applicable
**WDup**	0.753	0.325	not applicable	not applicable

Pelvic movement asymmetry parameters: PDmin, PDmax, PDup, HHD; head movement asymmetry parameters: HDmin, HDmax, HDup; withers movement asymmetry parameters WDmin, WDmax, WDup; orthotic lift conditions: ‘no lift’, L15, R15, L30, R30 describing unilateral lifts of 15 and 30mm applied to the left (L) or right (R) hind limb. Sample size: N = 11 for ‘no lift’, L30 and R30; N = 10 for L15 and R15

**Table 2:** Estimated marginal means (and 95% confidence intervals) from mixed models for movement asymmetry parameters with orthotic lift condition as fixed effect, stride time as covariate and horse as random effect. Directions of asymmetry parameters are marked with L or R depending on whether the direction of asymmetry would be found in horses with left (L) or right (R) sided lameness: the directional relationship is indicated in the column ‘direction’ linking positive (+ve) or negative (-ve) values to L-sided or R-sided asymmetry.

param	Direction	‘no lift’	L15	R15	L30	R30
**PDmin**	+ve L, -ve R	0.6L [4.6R 5.8L]	6.8L [1.5L 12.1L]	2.8R [8.1R 2.6L]	9.3L [4.0L 14.5L]	10.0R [15.3R 4.8R]
**PDmax**	+ve R, -ve L	2.2R [3.7L 8.1R]	5.9R [0 11.9R]	3.4L [9.4L 2.5R]	3.5R [2.4L 9.4R]	5.8L [11.7L 0.1R]
**PDup**	+ve R, -ve L	1.6R [4.9L 8.1R]	0.8L [7.5L 5.8R]	0.6L [7.3L 6.0R]	5.8L [12.3L 0.8R]	4.2R [2.3L 10.8R]
**HHD**	+ve L, -ve R	2R [9.1R 5.0L]	6.2L [1.2R 13.5L]	0 [7.4R 7.3L]	3.1L [4.3R 10.4L]	0.1R [7.1R 7.0L]
**HDmin**	+ve R, -ve L	0.6R [9.7L 10.9R]	2.0R [8.3L 12.4R]	1.3L [11.7L 9.0R]	1.2R [9.2L 11.5R]	2.9L [13.2L 7.4R]
**HDmax**	+ve L, -ve R	0.6L [6.4R 7.6L]	2.1L [5.0R 9.1L]	2.9L [4.1R 10.0L]	1.1R [8.1R 5.9L]	8.7L [1.7L 15.7L]
**HDup**	+ve L, -ve R	0.1R [14.7R 14.6L]	0 [14.7R 14.7L]	4.3L [10.5R 19.0L]	2.2R [16.9R 12.5L]	11.6L [3.1R 26.3L]
**WDmin**	+ve R, -ve L	1.7L [6.7L 3.3R]	0.1R [5.0L 5.1R]	4.4L [9.4L 0.7R]	0.1L [5.1L 4.9R]	6.2L [11.2L 1.2L]
**WDmax**	+ve L, -ve R	1.4L [4.8R 7.7L]	1.4L [4.8R 7.7L]	2.1R [8.3R 4.2R]	1.6L [4.6R 7.9L]	1.4R [7.6R 4.8L]
**WDup**	+ve L, -ve R	3.1L [5.5R 11.7L]	1.4L [7.3R 10.0L]	2.3L [6.3R 11.0L]	1.7L [6.9R 10.3L]	4.8L [3.8R 13.3L]

All values are given in mm.

**Fig 2:**
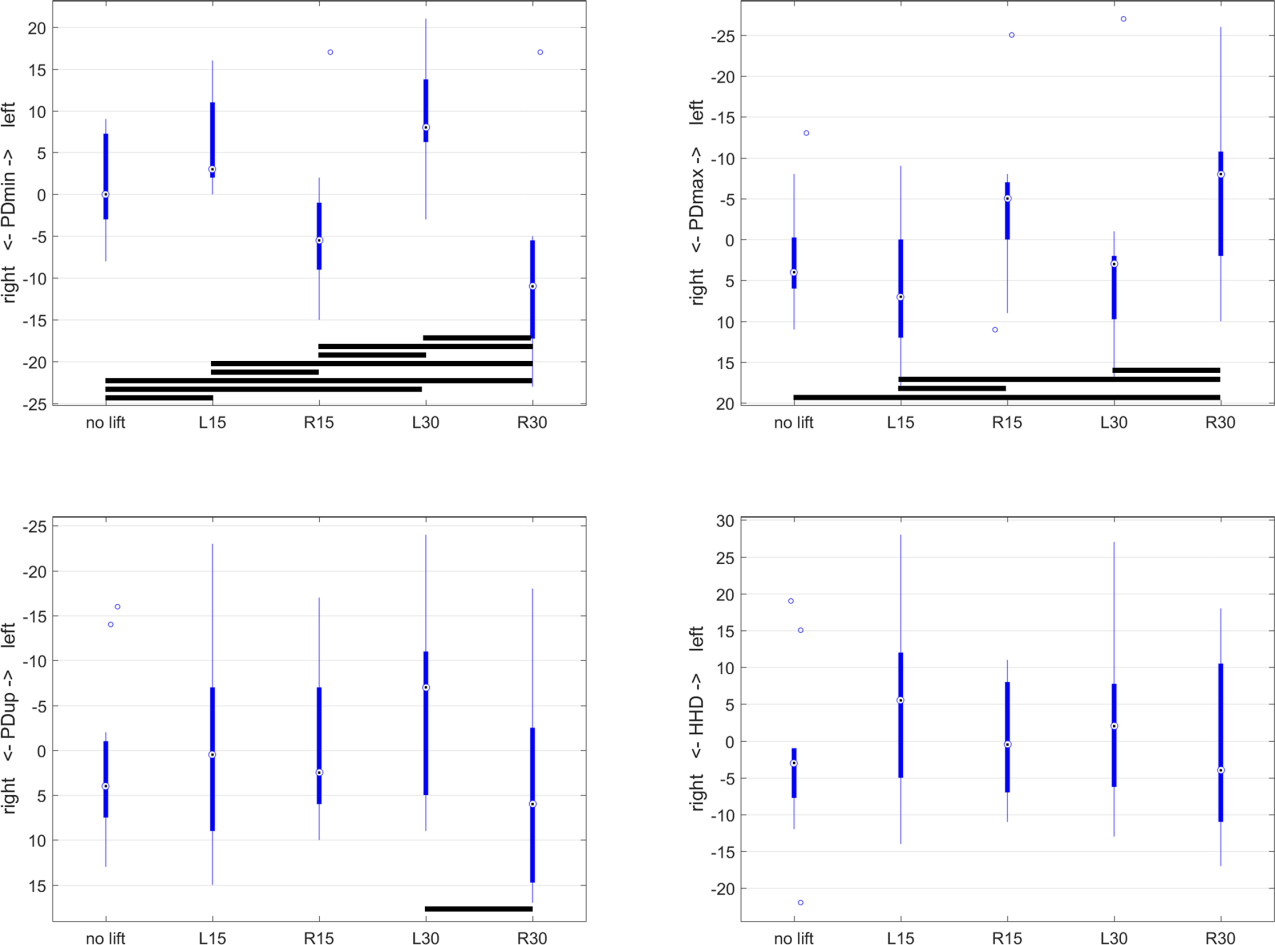
Boxplots for pelvic movement asymmetry parameters as a function of orthotic lift conditions (‘no lift’ and 15mm (L15 and R15) and 30mm (L30 and R30) left and right unilateral orthotic hind limb lifts). A: PDmin, B: PDmax, C: PDup, D: HHD. Pairwise significant differences (Bonferroni corrected P-values in Table 1) are indicated in each box plot by black horizontal lines between the conditions for which significant differences were found.

Pelvic movement asymmetry was found to be significantly influenced by the application of unilateral orthotic hind limb lifts with the mixed linear model for PDmin (P<0.001), PDmax (P<0.001) and PDup (P = 0.020) but not for HHD (P = 0.348), indicating a significant influence of orthotic lift condition on movement asymmetry of the tubera sacrale, but not of the difference between left and right tubera coxae. PDmin showed a change in movement asymmetry consistent with a reduction in weight bearing with the limb ipsilateral to the fitted orthotic hind limb lift, i.e. left-sided asymmetry for left-sided lifts and right-sided asymmetry for right-sided lifts (Table 2, Fig 2). PDmax showed a change in movement asymmetry consistent with a reduction in pushoff with the limb contralateral to the fitted orthotic lift, i.e. a right-sided asymmetry for left-sided lifts and left-sided asymmetry for right-sided lifts. PDup showed one significant pairwise difference between the two 30mm lifts (30L and 30R).

Estimated marginal means for PDmin were 7 to 9mm for left-sided lifts and 3 to 10mm for right sided lifts (Table 2). For PDmax for left-sided lifts, 4 to 6mm asymmetry were found and 3 to 6mm for right-sided lifts (Table 2). PDup showed 6mm left-sided asymmetry for L30 and 4mm right-sided asymmetry for R30.

**Fig 3:**
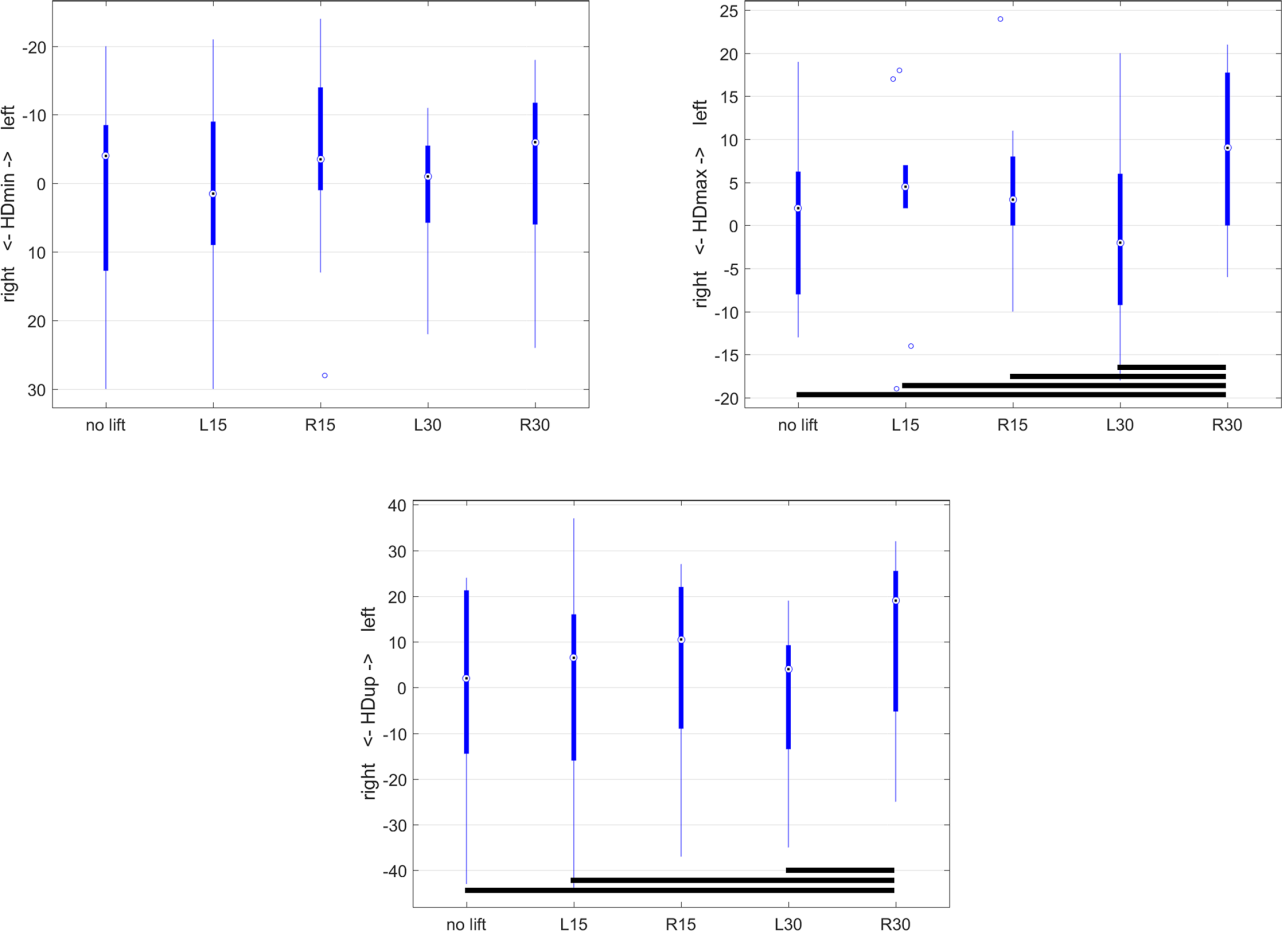
Boxplots for head movement asymmetry parameters as a function of orthotic lift conditions (‘no lift’ and 15mm (L15 and R15) and 30mm (L30 and R30) left and right unilateral orthotic hind limb lifts). A: HDmin, B: HDmax, C: HDup. Pairwise significant differences (Bonferroni corrected P-values in Table 1) are indicated in each box plot by black horizontal lines between the conditions for which significant differences were found.

Head movement asymmetry was found to be significantly influenced by the application of unilateral orthotic hind limb lifts with the mixed linear model for HDmax (P<0.001) and HDup (P<0.001) but not for HDmin (P = 0.096) indicating a significant influence of condition for 2 out of three head movement symmetry parameters (Table 1). Pairwise comparisons for HDmax revealed that condition R30 was significantly different to all other conditions and, in particular, created the most left-sided asymmetry of all conditions (8.7mm left-sided, Table 2) while the only right-sided value for HDmax was found for L30 (1.1mm right-sided, Table 2). A similar trend was found for HDup. Illustrations of the data distribution for head movement asymmetry across the 5 conditions are given in Fig 3.

**Fig 4:**
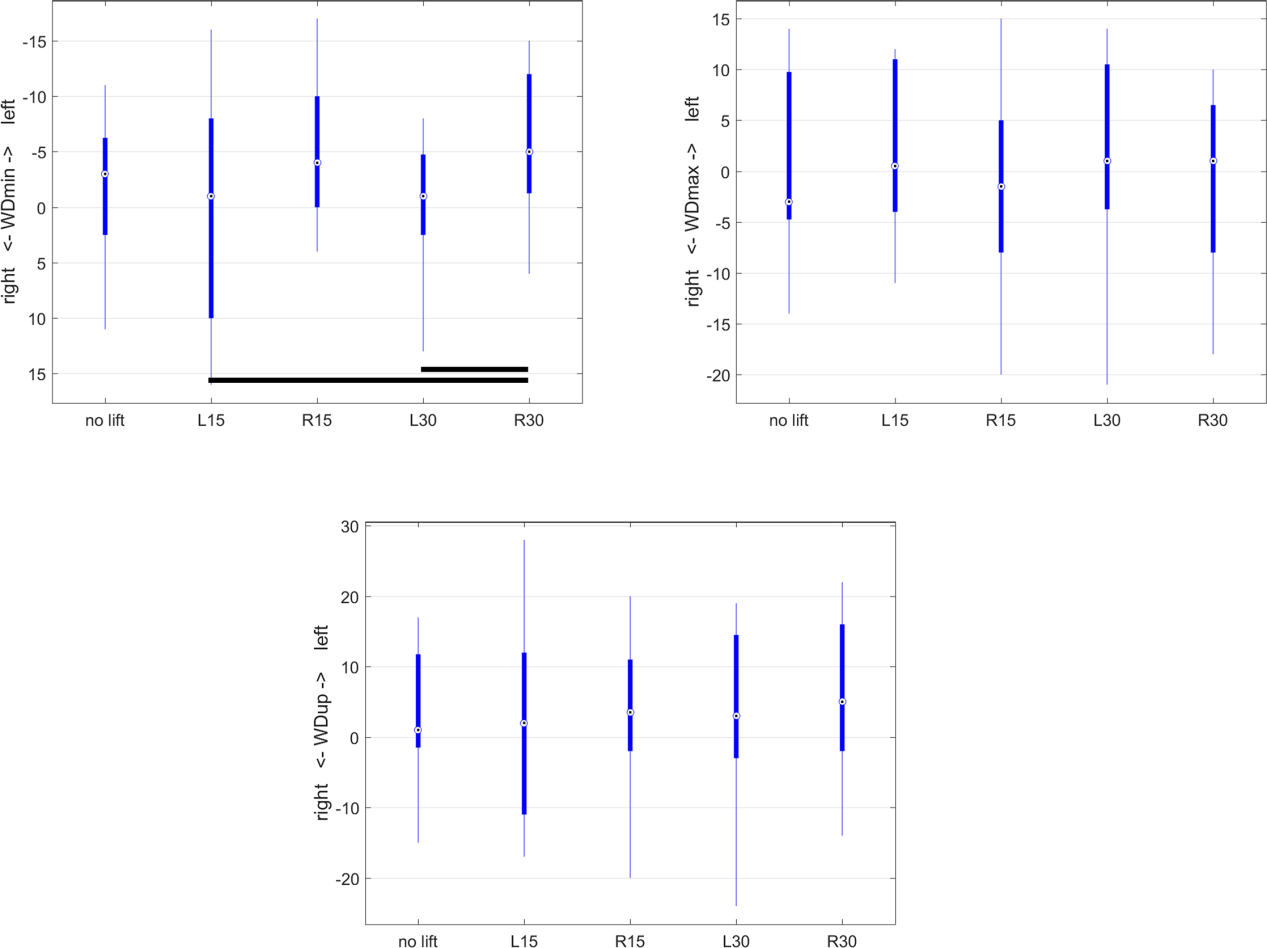
Boxplots for withers movement asymmetry parameters as a function of orthotic lift conditions (‘no lift’ and 15mm (L15 and R15) and 30mm (L30 and R30) left and right unilateral orthotic hind limb lifts). A: WDmin, B: WDmax, C: WDup. Pairwise significant differences (Bonferroni corrected P-values in Table 1) are indicated in each box plot by black horizontal lines between the conditions for which significant differences were found.

The only withers movement asymmetry parameter found to be significantly influenced by orthotic lift condition was WDmin (P<0.003). WDmax (P = 0.102) and WDup (P = 0.753) were not influenced by lift condition (Table 1). WDmin showed the most left-sided asymmetry for R30 (more left-sided than the two left-sided lifts) with an estimated marginal mean value of 6mm (Table 2). Fig 4 illustrates the data distribution of withers movement asymmetry across the 5 conditions.

## Discussion

This study has investigated the application of an artificial lengthening of a pelvic limb–a simple mechanical change–termed an orthotic lift and its immediate influence on pelvic as well as head and withers movement asymmetry parameters. The most consistent changes were measured for pelvic movement asymmetry and these are in agreement with the effects observed in humans [20,21]. Both head and withers movement asymmetry were also affected indicating consequences expanding across the whole trunk and head-neck segment.

### Pelvic movement asymmetry and unilateral orthotic hind limb lift

Pelvic movement asymmetry showed opposite-sided effects for PDmin and PDmax. While PDmin is commonly associated with peak force asymmetry between left and right hind limbs, PDmax is related to differences in the propulsive effort in the second half of stance as well as implicated in the transfer between vertical and horizontal impulse [4]. Horses with induced hind limb lameness (using a validated lameness model [28]) show a different pattern than the horses in this study: same-sided effect for PDmin and PDmax i.e. a reduction in both downward and upwards movement of the pelvis during the stance phase of the lame limb [3]. That pattern is also found in many (not all) clinically hind limb lame horses [6,13]. The opposite-sided changes in PDmin and PDmax found here suggest that horses move mechanically differently immediately after being fitted with an orthotic lift–an artificial lengthening of one of the hind limbs–compared to an induced (painful) lameness [14] or indeed differently to common patterns seen in naturally asymmetrical horses [29].

Whether or not the prediction of force asymmetry from upper body kinematics, as found previously [4,5], works reliably in the presence of limb length discrepancies ultimately needs clarification by means of concurrent measurement of ground reaction force. Studies in human subjects with natural and artificial limb length discrepancies provide initial support for the ability to predict force asymmetry from kinematic asymmetries even under these circumstances: there the shorter limb experiences a higher peak vertical force and the longer limb provides more propulsion [20,21]. This would also be predicted from our study: with a left-sided orthotic lift (longer LH limb) a horse appears left-asymmetrical in terms of PDmin, i.e. shows a pattern commonly seen in a LH lame horse, which produces more force with the contralateral RH (here: shorter) limb [10]. With the same left-sided orthotic lift, a horse appears more right-asymmetrical in terms of PDmax, i.e. shows a reduction in propulsive effort with the (shorter) RH limb compared to the (longer) LH limb again agreeing with the force effects in humans.

Opposite effects in terms of directional changes in PDmin and PDmax may also help explain why no consistent effect was measured for HHD. HHD is a lameness indicator used when visually identifying hind limb lameness and compares the vertical movement of the left to the right tuber coxae [2]. Assuming, that in a horse with a left-sided hind limb lift, PDmin is associated with reduced weight bearing with the LH limb (a higher position of the pelvis during LH stance related to a reduction in fetlock hyperextension as a result of the linear relationship between force and fetlock joint angle [30]) and PDmax is associated with a reduced pelvic height reached after RH limb stance, the upward movement asymmetry (the difference between the maximum and the minimum position) of either the tubera coxae (expressed as HHD) or of the midline of the pelvis (expressed as PDup) would be expected to remain unaffected by the opposing shift in force.

This effect may both be a blessing and a curse for visual assessment of horses, the former since when assessing lameness by comparing left to right tuber coxae movement asymmetry, a horse with a limb length discrepancy and no underlying painful pathology (which presumably would cause a typical hip hike [2]) may not be categorized as lame visually. The latter, since observers visually assessing downward movement or upward movement symmetry of the mid pelvis (rather than tubera coxae movement) may come to different conclusions, however a subsequently initiated clinical lameness exam with diagnostic analgesia should then reveal that no painful condition is present. The fact that movement patterns can show mid-pelvic asymmetry but no asymmetry at the level of the tubera coxae may in fact contribute to the poor inter-observer agreement between experts in horses with mild hind limb lameness [31].

### Head and withers movement asymmetry

Pelvic movement asymmetry showed the highest number of pairwise significant differences in relation to the use of the hind limb lifts. This is not surprising given the direct association between hind limb forces and pelvic movement asymmetry [4]. However, both head and withers movement asymmetry were also affected. This appears to be of relevance in the context of compensatory mechanisms. It is widely accepted (and referred to as the ‘rule of sides’ [13]) that horses with clinical hind limb lameness show changes in head movement–mimicking the pattern of a forelimb lameness ipsilateral to the hind limb lameness–and horses with clinical forelimb lameness show altered pelvic movement–mimicking the pattern of a hind limb lameness contralateral to the forelimb lameness [12,13]. Recent findings show, that hind limb lameness (specifically its elimination) has consistent effects along the thoracolumbosacral region [32] and that withers movement appears to be of discriminative quality in the context of induced forelimb and hind limb lameness [14].

In our study, head movement asymmetry showed a tendency towards more left-sided (LF) asymmetry for right-sided hind limb lifts and vice versa. For example, HDmax showed the most left-sided asymmetry for R30 and the most right-sided asymmetry for L30. This is the opposite pattern to PDmin (left-sided for L15/L30, right-sided for R15/R30) but agrees with the sidedness of PDmax (left-sided for R15/R30, right-sided for L15/L30). The fact that PDmin and PDmax are showing opposite-sided effects however complicates matters, since it is not immediately clear what the overall direction of pelvic movement asymmetry is: a left-sided lift creates the appearance of a mixed LH weight bearing and RH pushoff asymmetry. It would be interesting to further study the (compensatory) head and withers movement effect in horses with clinically diagnosed lameness and for example contrast the effects of the use of an orthotic lift on the straight to effects seen on the lunge [33]. Lungeing seems to have similar effects: reduced weight bearing with the inside hind leg, reduced pushoff with the outside hind leg. The inward lean of a horse on the lunge effectively lengthens the inside leg and shortens the outside leg and hence the ‘longer’ inside leg would be expected to experience less force and the shorter outside leg would be expected to create less pushoff [20,21]. However, intriguingly on the lunge horses also show a consistent increase in inside tuber coxae movement amplitude [34] which makes lungeing different from the use of an orthotic lift on the straight. Finally, while not significantly affected by lift condition, WDmax appears to show a trend of counteracting HDmax in direction, indicating that this may be a compensatory mechanism (based on the observation of opposing head and withers asymmetry in induced hind limb lameness [14]), likely in reaction to the changes in PDmax, which agree in direction with changes in HDmax and hence agree with the ‘rule of sides’ for hind limb lameness.

### Practical relevance

Our findings that artificial limb length discrepancies are influencing movement asymmetry (immediately after application) have practical consequences.

First, a horse presenting with a small and consistent PDmin value suggesting a LH lameness and a small and consistent PDmax value suggesting a RH lameness may in fact not experience pain in either the LH or RH limb. It is possible that (at least part of) the movement asymmetry pattern is related to a mechanical difference that could be addressed by changes to trimming and shoeing, for which small changes in movement asymmetry have been observed [35]. The term ‘mechanical lameness’ springs to mind here. The size of the induced pelvic movement asymmetries of 3 to 6mm even for the smaller 15mm lifts (L15, R15) indicates that a limb length discrepancy between hind limbs should not be neglected as a contributing factor in further veterinary investigations after having screened a horse for lameness by use of gait analysis. These asymmetry values are similar to previously reported thresholds of 3mm for pelvic movement asymmetry and 6mm for head movement asymmetry; in both cases it appears essential in addition to the amount of asymmetry to also assess its variability, e.g. comparing the mean value over a sufficiently high number of strides to the standard deviation across strides [26].

Second, and related to the first point, the influence of limb length discrepancies in both the development of pathology as well as the rehabilitation from orthopaedic deficits might need further investigation. Our study suggests that small limb length discrepancies immediately lead to asymmetry of force production which, if persistent over longer time periods, may lead to overuse injuries. Hence, the aspect of persistence of limb length related movement asymmetries over days or weeks after application of an orthotic lift seems worthy of further investigation. When rehabilitating a horse after correction of an orthopaedic problem, it is possible, that the horse shows chronic changes in hoof shape. If left unattended, our results suggest that without addressing these changes, and assuming the short-term effects measured here persist over longer time periods, the difference in limb length (hoof height) may prevent the horse from going back to perfectly symmetrical movement and likely force production.

### Limitations

The sample size of this study was small, limiting the generalizability of our results. The study design, investigating immediate changes within horses with subsequent left and right sided unilateral interventions aimed to reduce the influence of inter-horse variability. The persistence of the measured effects over days or weeks was not assessed in this study and as such this limits any conclusions drawn about potential long-term consequences for example in the context of rehabilitation. This aspect needs further investigation.

We have only measured upper body kinematics and no direct force measurements were conducted. Hence, while the measured effects agree with force changes measured in bipeds, it remains to be proven, that the kinematics accurately predict force asymmetry when manipulating limb length. However, based on first principles, like the mathematical relationship between displacement and velocity and velocity and acceleration and Newtonian Physics (F = m*a), a reduction in vertical movement during one stance phase compared to the other would appear to be related to a reduction in force production during that stance phase further supporting any conclusions referring to force distribution.

In an attempt to limit the effects of altered mass between contralateral limbs, the shoe attached to the bottom of the contralateral boot (the one without the orthotic lift) in the 30mm condition, meant that the effective height difference on a hard surface would only have been 23mm between limbs. How big the effective height difference will be on average across strides on the soft surfaces used here was impossible to ascertain in the current study. This however should be further investigated in future studies, e.g. investigating the effects during lungeing.

## Supporting information

S1 Table Raw data. Given are for each horse and each available orthotic lift condition (cond.) values (median values over all strides per condition) for all ten movement asymmetry variables as well as number of strides per condition (# strides) and average stride time per condition (time). Average values, minima, maxima and sum (number of strides) are given at the bottom of the table. All movement asymmetry values in mm.
(DOCX)
