# Supplementary material for: Effect of a unilateral hind limb orthotic lift on upper body movement symmetry in the trotting horse
Source: PLoS One. 2018 Jun 21;13(6):e0199447. doi: 10.1371/journal.pone.0199447 (PMC6013171; doi:10.1371/journal.pone.0199447)
Supplement: S1 Table — Given are for each horse and each available orthotic lift condition (cond.) values (median values over all strides per condition) for all ten movement asymmetry variables as well as number of strides per condition (# strides) and average stride time per condition (time). Average values, minima, maxima and sum (number of strides) are given at the bottom of the table. All movement asymmetry values in mm. (DOCX) [file pone.0199447.s001.docx]

Table S1: Raw data. Given are for each horse and each available orthotic lift condition (cond.) values (median values over all strides per condition) for all ten movement asymmetry variables as well as number of strides per condition (# strides) and average stride time per condition (time). Average values, minima, maxima and sum (number of strides) are given at the bottom of the table. All movement asymmetry values in mm.

| **horse ID** | **cond.** | **# strides** | **time (ms)** | **HD min** | **HD max** | **HD up** | **WD min** | **WD max** | **WD up** | **PD min** | **PD max** | **PD up** | **HHD** |
| --- | --- | --- | --- | --- | --- | --- | --- | --- | --- | --- | --- | --- | --- |
| **1** | **no lift** | **23** | **748** | **-5** | **19** | **24** | **-3** | **9** | **12** | **-2** | **6** | **8** | **-2** |
| 1 | L15 | 23 | 745 | 4 | 18 | 14 | -1 | 11 | 12 | 3 | 12 | 9 | -5 |
| 1 | R15 | 22 | 739 | -3 | 24 | 27 | -3 | 5 | 8 | -9 | -5 | 4 | 3 |
| 1 | L30 | 19 | 749 | 1 | 20 | 19 | -1 | 14 | 15 | 8 | 16 | 8 | -4 |
| 1 | R30 | 21 | 728 | -10 | 21 | 31 | -1 | 1 | 2 | -13 | -11 | 2 | 9 |
| **2** | **no lift** | **16** | **631** | **0** | **2** | **2** | **-3** | **-3** | **0** | **-3** | **-1** | **2** | **-3** |
| 2 | L15 | 32 | 653 | 5 | 2 | -3 | -4 | -1 | 3 | 0 | 4 | 4 | 4 |
| 2 | R15 | 29 | 630 | 1 | 7 | 6 | -5 | -6 | -1 | -4 | -7 | -3 | 9 |
| 2 | L30 | 30 | 646 | 5 | 2 | -3 | -2 | 1 | 3 | 9 | 2 | -7 | 12 |
| 2 | R30 | 32 | 644 | -4 | 14 | 18 | -5 | -8 | -3 | -11 | -12 | -1 | 9 |
| **5** | **no lift** | **28** | **655** | **17** | **10** | **-7** | **1** | **-14** | **-15** | **1** | **-13** | **-14** | **15** |
| 5 | L15 | 19 | 656 | 20 | 4 | -16 | 0 | -11 | -11 | 6 | -9 | -15 | 12 |
| 5 | R15 | 26 | 636 | 13 | 4 | -9 | 0 | -20 | -20 | -8 | -25 | -17 | -4 |
| 5 | L30 | 26 | 638 | 18 | 3 | -15 | 3 | -21 | -24 | -3 | -27 | -24 | 27 |
| 5 | R30 | 17 | 624 | 10 | 13 | 3 | -5 | -18 | -13 | -19 | -26 | -7 | 12 |
| **7** | **no lift** | **36** | **701** | **30** | **-13** | **-43** | **8** | **-5** | **-13** | **8** | **6** | **-2** | **-3** |
| 7 | L15 | 36 | 692 | 30 | -14 | -44 | 10 | -7 | -17 | 13 | 6 | -7 | 9 |
| 7 | R15 | 38 | 692 | 28 | -9 | -37 | 4 | -6 | -10 | 2 | 11 | 9 | -6 |
| 7 | L30 | 32 | 690 | 22 | -13 | -35 | 13 | -9 | -22 | 21 | 3 | -18 | NA |
| 7 | R30 | 34 | 689 | 24 | -1 | -25 | 0 | 10 | 10 | -5 | 3 | 8 | -6 |
| **8** | **no lift** | **27** | **681** | **13** | **-10** | **-23** | **-4** | **-6** | **-2** | **-8** | **2** | **10** | **-9** |
| 8 | L15 | 31 | 676 | 9 | -19 | -28 | -1 | -4 | -3 | 2 | 8 | 6 | -8 |
| 8 | R15 | 24 | 688 | 1 | -10 | -11 | -7 | -9 | -2 | -15 | -5 | 10 | -7 |
| 8 | L30 | 27 | 695 | 6 | -18 | -24 | -1 | -1 | 0 | 4 | 10 | 6 | -9 |
| 8 | R30 | 23 | 714 | 3 | -5 | -8 | -12 | -11 | 1 | -23 | -8 | 15 | -12 |
| **9** | **no lift** | **32** | **727** | **12** | **-5** | **-17** | **-4** | **-3** | **1** | **5** | **10** | **5** | **-12** |
| 9 | L30 | 31 | 727 | -1 | -10 | -9 | 1 | -3 | -4 | 13 | 2 | -11 | 2 |
| 9 | R30 | 35 | 740 | 7 | -6 | -13 | 6 | -8 | -14 | 17 | -1 | -18 | 18 |
| **10** | **no lift** | **26** | **630** | **-19** | **4** | **23** | **-10** | **7** | **17** | **-3** | **3** | **6** | **-4** |
| 10 | L15 | 23 | 636 | -20 | 17 | 37 | -16 | 12 | 28 | 3 | 18 | 15 | -14 |
| 10 | R15 | 23 | 643 | -5 | 2 | 7 | -17 | 3 | 20 | -5 | -3 | 2 | 4 |
| 10 | L30 | 22 | 642 | -4 | 9 | 13 | -8 | 11 | 19 | 8 | 17 | 9 | -13 |
| 10 | R30 | 24 | 645 | -13 | 19 | 32 | -13 | 5 | 18 | -15 | -9 | 6 | -4 |
| **11** | **no lift** | **28** | **688** | **-20** | **4** | **24** | **3** | **14** | **11** | **9** | **11** | **2** | **-1** |
| 11 | L15 | 31 | 678 | -21 | 7 | 28 | 10 | 11 | 1 | 11 | 8 | -3 | 3 |
| 11 | R15 | 29 | 683 | -24 | 2 | 26 | 4 | 15 | 11 | 17 | 9 | -8 | 8 |
| 11 | L30 | 28 | 715 | -8 | -4 | 4 | -6 | 13 | 19 | 16 | 9 | -7 | 6 |
| 11 | R30 | 21 | 696 | -18 | 3 | 21 | -10 | 9 | 19 | -5 | 10 | 15 | -17 |
| **12** | **no lift** | **22** | **805** | **-7** | **-3** | **4** | **-11** | **-4** | **7** | **-8** | **5** | **13** | **-22** |
| 12 | L15 | 20 | 782 | -9 | 7 | 16 | -8 | 0 | 8 | 1 | 15 | 14 | 28 |
| 12 | R15 | 13 | 790 | -14 | 8 | 22 | -10 | -8 | 2 | -9 | -6 | 3 | -11 |
| 12 | L30 | 22 | 803 | -6 | -2 | 4 | -5 | -4 | 1 | 7 | 9 | 2 | -7 |
| 12 | R30 | 22 | 803 | -12 | 7 | 19 | -12 | -5 | 7 | -18 | -4 | 14 | -11 |
| **13** | **no lift** | **24** | **695** | **-4** | **-9** | **-5** | **11** | **11** | **0** | **8** | **-8** | **-16** | **19** |
| 13 | L15 | 26 | 703 | -4 | 3 | 7 | 16 | 1 | -15 | 16 | -7 | -23 | 26 |
| 13 | R15 | 26 | 710 | -14 | 0 | 14 | -1 | 4 | 5 | -1 | -8 | -7 | 11 |
| 13 | L30 | 27 | 704 | -11 | -7 | 4 | 6 | 9 | 3 | 14 | 3 | -11 | 8 |
| 13 | R30 | 28 | 703 | -11 | 9 | 20 | -2 | 3 | 5 | -7 | -10 | -3 | 11 |
| **14** | **no lift** | **25** | **708** | **-9** | **7** | **16** | **-7** | **10** | **17** | **0** | **4** | **4** | **-1** |
| 14 | L15 | 22 | 691 | -1 | 5 | 6 | -8 | 7 | 15 | 3 | 0 | -3 | 7 |
| 14 | R15 | 23 | 698 | -4 | 11 | 15 | -11 | 6 | 17 | -6 | 0 | 6 | -7 |
| 14 | L30 | 22 | 709 | -4 | 7 | 11 | -4 | 9 | 13 | 6 | -1 | -7 | 7 |
| 14 | R30 | 19 | 697 | -6 | 21 | 27 | -15 | 7 | 22 | -11 | 6 | 17 | -11 |
|  | **average** | **25.8** | **696.1** | **-0.2** | **3.0** | **3.2** | **-2.6** | **0.4** | **3.1** | **0.4** | **0.4** | **0.0** | **1.3** |
|  | **min** | **13** | **624** | **-24** | **-19** | **-44** | **-17** | **-21** | **-24** | **-23** | **-27** | **-24** | **-22** |
|  | **max** | **38** | **805** | **30** | **24** | **37** | **16** | **15** | **28** | **21** | **18** | **17** | **28** |
|  | **sum** | **1365** | **NA** | **NA** | **NA** | **NA** | **NA** | **NA** | **NA** | **NA** | **NA** | **NA** | **NA** |
